# Emerging targeted therapies for primary immune thrombocytopenia: novel agents, combination strategies, and future directions

**DOI:** 10.3389/fphar.2026.1924052

**Published:** 2026-08-27

**Authors:** Zhen Wang, Wuxia Yang, Nuo Xu, Lin Ma, Aidi Wang, Baoshan Liu

**Affiliations:** 1 Tianjin Medical University General Hospital, Tianjin, China; 2 Graduate School, Tianjin University of Traditional Chinese Medicine, Tianjin, China; 3 Graduate School, Tianjin Medical University, Tianjin, China

**Keywords:** autoimmunity, clinical trials, potential pathogenesis, primary immune thrombocytopenia, therapeutic drugs

## Abstract

**Background:**

An autoimmune disease with notable clinical variability, primary immune thrombocytopenia is typified by immune-mediated platelet underproduction and destruction. Conventional therapies, including glucocorticoids and splenectomy, are limited by adverse effects, high relapse rates, and surgical risks. Recently, with the in-depth study of immune thrombocytopenia, new targeted drugs have emerged rapidly.

**Methods:**

We conducted a systematic literature search of PubMed, Web of Science, and ClinicalTrials.gov from May 2010 up to May 2026, using search terms related to “immune thrombocytopenia” AND “targeted therapy” OR “novel agents.” Eligible studies included phase I–III clinical trials, prospective observational studies, and high-quality preclinical mechanistic studies evaluating novel ITP therapeutics, while case reports and editorials were excluded. Two independent reviewers screened titles and abstracts, followed by full-text assessment, with discrepancies resolved by consensus. Given the heterogeneous nature of the included evidence, we adopted a narrative synthesis approach, complemented by a clinical evidence-grading framework that stratified emerging agents by the maturity of their supporting data (phase I, phase II, phase III, or preclinical studies), rather than performing a quantitative meta-analysis.

**Conclusion:**

This article analyses recent therapeutic trials with Bruton’s tyrosine kinase inhibitors, splenic tyrosine kinase inhibitors, and neonatal Fc receptor antagonists, demonstrating an improvement in platelet counts and patients’ quality of life. Furthermore, combination therapy regimens show great promise in terms of synergistic effects, while cellular therapies, such as chimeric antigen receptor T-cell immunotherapy and mesenchymal stem cell therapy, appear to offer new treatment concepts for patients with persistent, chronic, refractory, or multi-refractory ITP. However, there are still many problems and shortcomings in the diagnosis and treatment of immune thrombocytopenia. In addition, the transition from preliminary evidence to routine clinical application requires larger phase III confirmatory studies, validated biomarker-driven patient selection, and context-adapted treatment algorithms that address resource-limited settings. Future research should prioritize head-to-head comparisons, combination regimens, and real-world effectiveness studies to optimize long-term outcomes for all ITP patients.

## 1 Introduction

Primary immune thrombocytopenia (ITP) is an acquired autoimmune illness that causes isolated thrombocytopenia and an increased risk of bleeding (Bu et al., 2025). The main cause of the disease is an imbalance in the body’s immune tolerance, leading B cells to produce anti-platelet autoantibodies, such as anti-Glycoprotein (GP) IIb/IIIa and anti-GPIb/IX complexes. These autoantibodies accelerate platelet destruction via Fc receptor-mediated phagocytosis by macrophages and block megakaryocyte differentiation and platelet production, creating a “double attack” pattern that is harmful (Mititelu et al., 2024; Li et al., 2024). The global incidence of ITP is between 2 and 5 per 100,000. Approximately 30% of patients develop chronic or refractory ITP with clinical presentations varying significantly, ranging from asymptomatic to catastrophic visceral bleeding, with large individual variances (Neunert et al., 2024; Gallo and Lambert, 2024).

The traditional standard of care is a steroid hormone and splenectomy. Short-term platelet counts improve in 60%–80% of patients with first-line glucocorticoid therapy, such as prednisone. However, long-term use raises the risk of infection, osteoporosis, and metabolic syndrome, and 50% of patients relapse after reducing their dosage or stopping it (Frederiksen, 2024; Wang et al., 2024). Although splenectomy was once the ‘gold standard’ for second-line therapy, its invasive nature and 20%–30% relapse rate have reserved it for patients who fail or cannot tolerate medical treatments (Rabinovich et al., 2024). Because of these problems, doctors have had to rethink the therapeutic goals of ITP. They are no longer just focused on keeping patients’ platelet counts up. Instead, they are trying to improve patients’ quality of life by reducing fatigue, preventing treatment side effects, and providing long-term, personalised care. In addition, intravenous immunoglobulin, rituximab, fostamatinib, and other treatment options are also available for many ITP patients.

**FIGURE 1:**
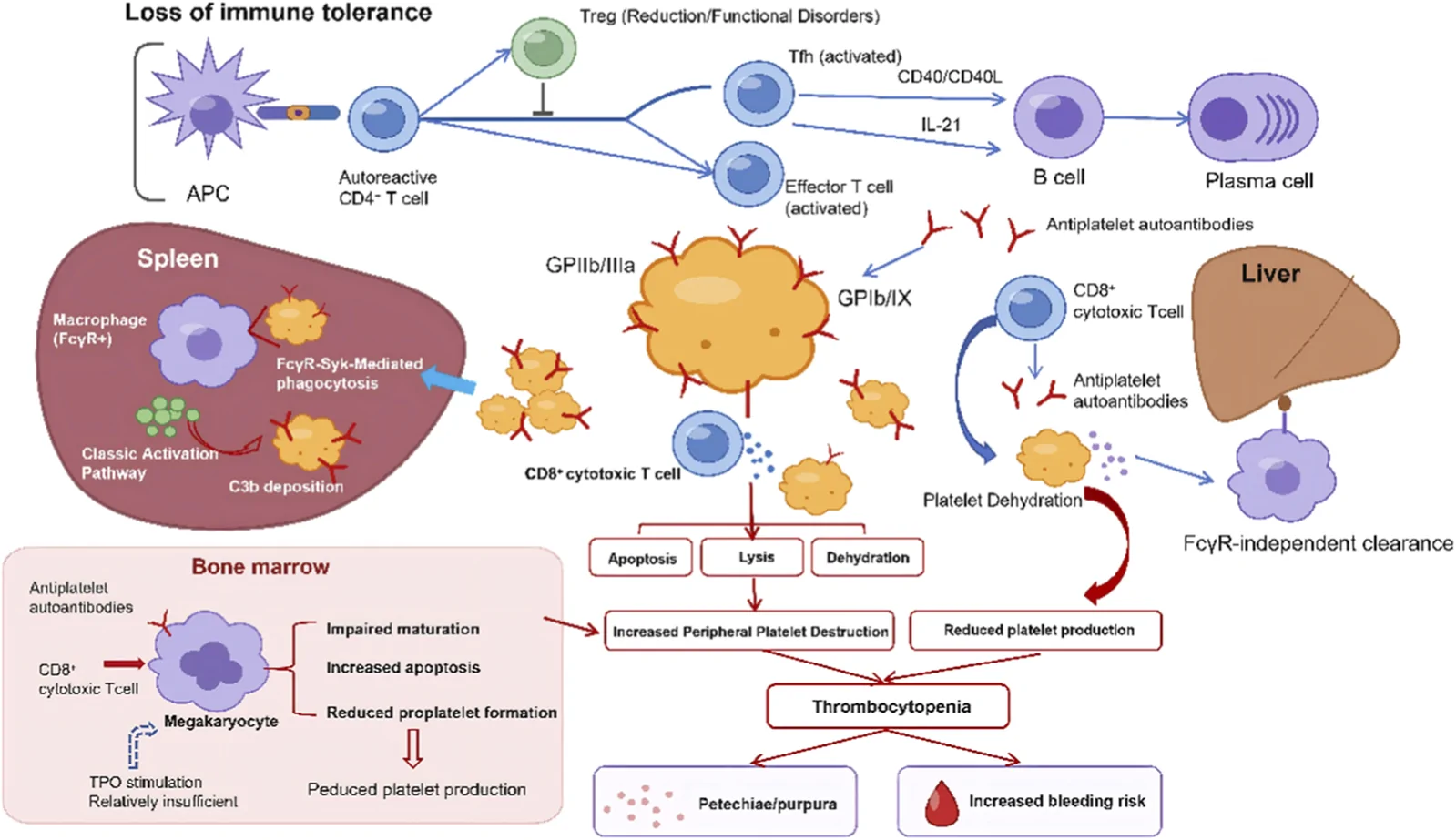
The potential pathogenesis of immune thrombocytopenia. This schematic depicts the multifaceted pathogenic pathways by which breakdown of immune tolerance leads to thrombocytopenia. In secondary lymphoid organs, autoreactive CD4^+^ T cells are primed by antigen-presenting cells and differentiate into follicular helper T cells and effector T cells. Tfh cells support B-cell maturation into plasma cells via CD40/CD40L interaction and IL-21 secretion. Plasma cells produce anti-platelet autoantibodies that target glycoprotein GPIIb/IIIa and GPIb/IX complexes on the platelet surface. These autoantibodies mediate peripheral platelet destruction through two major routes in the spleen: (1) FcγR-Syk-dependent phagocytosis by FcγR^+^ macrophages, and (2) classic complement activation with C3b deposition, leading to lysis or enhanced clearance. In the liver, antibody-opsonized platelets undergo FcγR-independent clearance. Concurrently, in the bone marrow, anti-platelet autoantibodies and CD8^+^ cytotoxic T cells impair megakaryocyte maturation, increase apoptosis, and reduce both proplatelet and platelet formation. Although thrombopoietin stimulation persists, the resulting platelet production is relatively insufficient to offset peripheral losses. The combined decrease in production and increase in destruction culminate in thrombocytopenia, clinically manifesting as petechiae/purpura and an elevated risk of bleeding. Abbreviations: APC, Antigen-presenting cell; CD40, Cluster of differentiation 40; Tfh, Follicular helper T cell; FcγR, Fc gamma receptor; Syk, Spleen tyrosine kinase; TPO, Thrombopoietin; GPIIb/IIIa, Glycoprotein IIb/IIIa; GPIb/IX, Glycoprotein Ib/IX complex.

In recent years, novel targeted therapeutic strategies have emerged with in-depth research on ITP. These discoveries, ranging from Thrombopoietin receptor agonists (TPO-RA) to neonatal Fc receptor (FcRn) inhibitors, Splenic tyrosine kinase (SYK) inhibitors, and disruptive Chimeric antigen receptor T-cell immunotherapy (CAR-T), have shed light on the treatment of adult patients with refractory ITP. Nonetheless, predicting therapeutic response, lowering post-treatment recurrence rates, and balancing safety and efficacy remain pressing issues. Distinct from existing ITP treatment reviews, our analysis not only summarizes emerging agents but critically appraises the strength of supporting evidence, explicitly distinguishing those supported by phase III data from those still in early-phase or preclinical investigation, while also examining combination strategies that have received limited systematic attention. Figure 1 shows the potential pathogenesis of immune thrombocytopenia.

## 2 Novel targeted therapies

### 2.1 TPO-RA

TPO-R (also known as c-Mpl or CD110) is a transmembrane protein that is mainly found on the surface of megakaryocytes, hematopoietic stem cells, and platelets. The combination of TPO-RA and TPO-R will activate downstream signalling pathways, promote megakaryocyte proliferation, differentiation, and maturation, and ultimately increase platelet production. For example, the JAK-STAT pathway, PI3K-AKT pathway, and MAPK pathway. Apart from promoting platelet formation, some TPO-RA (such as eltrombopag) also have immunomodulatory effects. They can increase the number of regulatory T cells, thereby alleviating autoimmune reactions, which is particularly crucial for ITP patients.

Romiplostim, a TPO-RA, increases platelet production by selectively binding to the dimerisation domain of the TPO receptor and triggering the downstream Janus kinase-signal transducer and activator of transcription signalling cascade. This boosts megakaryocyte proliferation and survival (Bussel et al., 2021). A Phase III clinical study in China (NCT02868099, CTR20150395) assessed the efficacy and tolerability of Romiplostim as a second-line treatment in adults with persistent or chronic ITP (Zhou et al., 2023a). There were 152 patients treated with Romiplostim and 51 treated with a placebo. During therapy, patients in the Romiplostim group had platelet counts ≥50 × 10^9^/L for an average of 2 weeks, compared to 0 weeks in the placebo group. At 22 weeks, 85.3% of patients in the Romiplostim group had platelet counts ≥20 × 10^9^/L, up from baseline. The safety profile of Romiplostim was consistent with previous studies, with similar rates of treatment-emergent adverse events (TEAEs) in the 2 groups (82.8% vs. 82.4%). This study partially validates Romiplostim as a second-line therapy for individuals with ITP.

Furthermore, avatrombopag is a TPO-RA that activates the thrombopoietin receptor by selectively binding to its transmembrane structural domain. This works like endogenous thrombopoietin, promoting megakaryocyte maturation and release to raise platelet counts and treat ITP (Lambert, 2024). A Spanish ITP research group conducted a retrospective real-world study to evaluate the efficacy and safety of avatrombopag in patients with ITP (n = 268) (Pascual-Izquierdo et al., 2024). Among patients with baseline platelet counts <50 × 10^9^/L, 90.1% (n = 174/193) achieved a therapeutic response (initial response, platelet counts ≥50 × 10^9^/L), while 97.3% (n = 73/75) of those with baseline platelet counts ≥50 × 10^9^/L reached a therapeutic response. Among patients who continued treatment (n = 129), 87.6% maintained their response through the end of the study. Neither the success nor the failure of prior TPO-RA(s) treatment affected the above results. Furthermore, avatrombopag enabled 18.6% (n = 50) of patients with baseline platelet counts <50 × 10^9^/L to discontinue or reduce their glucocorticoid use. Thrombocythemia (n = 40) and thrombotic embolic events (n = 12) were the most frequent treatment-related adverse events (TRAEs) associated with avatrombopag. In conclusion, while the AVESPA study stands as one of the largest real-world evaluations of avatrombopag to date with promising results, its retrospective, single-arm design introduces inherent biases that warrant cautious interpretation. Consequently, rigorously designed prospective studies are needed to further establish the long-term efficacy and safety of this agent.

QL0911 is a biosimilar of romiplostim with a mechanism of action similar to romiplostim. In April 2024, QL0911 received marketing approval in China for the treatment of persistent ITP in adults. The long-term effectiveness and safety of QL0911 in Chinese adult patients with chronic primary ITP at 24 weeks of treatment were confirmed by a phase III trial (NCT05621330) (Zhou et al., 2023b), which included 72 patients in the placebo arm and 144 patients in the QL0911 arm. At week 24 of treatment, 61.8% of patients in the QL0911 group had a durable platelet response (platelet counts ≥50 × 10^9^/L in 6 of the last 8 weeks), whereas none in the placebo group did. The mean duration of platelet response was significantly higher in the QL0911 group than in the placebo group (15.9 vs. 1.9 weeks). The most prevalent TEAEs were ecchymosis, upper respiratory tract infections, and gingival haemorrhage. According to the findings, QL0911 might develop into a novel treatment option for Chinese ITP patients who have relapsed or failed first-line therapy.

### 2.2 BTK inhibitors

Bruton’s tyrosine kinase (BTK) plays a key role in the B-cell receptor signalling pathway. By specifically blocking BTK activity, the oral BTK inhibitor rilzabrutinib reduces the formation of autoantibodies by preventing B-cell activation and proliferation (Langrish et al., 2021). Additionally, rilzabrutinib reduced the activation of inflammatory cells by blocking the IgG-mediated FcγR signalling pathway (Kuter et al., 2022).

LUNA2 Part B (NCT03395210) was a multicenter clinical trial that evaluated the clinical efficacy of rilzabrutinib in patients with ITP, focusing on the durability of platelet reactivity, and also conducting a comprehensive safety assessment (Cooper et al., 2025). A total of 26 patients were recruited, with an average of 6 prior ITP treatments; 15 completed the 24-week treatment. According to the results, 35% (n = 9/26) of patients reached the main goal of having platelet counts of 50 or more for at least 8 weeks without needing extra treatment during the last 12 weeks. Of the 11 (42%) qualifying patients who stayed on therapy during the long-term extension phase, the median platelet count was >80 × 10^9^/L. During the initial treatment period, 16 patients (62%) suffered at least one TRAE; grade 1 headache, nausea, and diarrhoea were the most common adverse events (AEs).

The Phase 3 LUNA 3 (NCT04562766) study (Kuter et al., 2025) assessed the efficacy and tolerability of rilzabrutinib versus placebo in patients with persistent or chronic ITP to determine whether rilzabrutinib can provide a sustained platelet response in these individuals. During the 24-week treatment period, 23% (n = 31/133) of patients who received rilzabrutinib achieved a durable platelet response, compared with none in the placebo group (n = 69). In addition, the rilzabrutinib group was significantly superior to the placebo group in all secondary efficacy endpoints. Safety evaluations revealed that the most common TRAEs in the rilzabrutinib group were grade 1 or 2 and were well tolerated by patients. The above results indicate that rilzabrutinib has the potential to be a new and effective treatment for ITP, providing additional options for patients who do not respond to existing therapies.

Orelabrutinib is also a BTK inhibitor. NCT05232149 was a Phase II trial (Yan S. et al., 2024) that investigated the effectiveness, safety, and tolerability of orelabrutinib in individuals with persistent or chronic primary ITP. 33 patients in all were recruited and randomly divided into two groups: 50 mg (n = 15) and 30 mg (n = 18). The primary endpoint of the trial was the percentage of participants who exhibited at least 2 consecutive platelet counts ≥50 × 10^9^/L within 4 weeks without requiring rescue medication. The study found that 36% (n = 12/33) of patients reached the primary endpoint, with 40% (n = 6/15) in the 50 mg arm and 22.2% (n = 4/18) in the 30 mg arm. Of those patients, 27.3% (n = 9/33) had at least 4 platelet counts ≥50 × 10^9^/L between weeks 14 and 24 of treatment and did not require rescue therapy in the 4 weeks preceding the rise in their platelet counts. No bleeding or thrombotic events were observed, and 24% of patients experienced treatment-related side effects, primarily grade 1 or 2. Urinary tract infections was frequent events (15%). Rilzabrutinib differs from orelabrutinib in its reversible BTK binding kinetics, which may theoretically confer a lower bleeding risk compared with irreversible inhibitors. However, the current evidence base—comprising a phase III trial for rilzabrutinib versus a phase II single-arm study for orelabrutinib, both with limited follow-up—precludes direct comparisons of their long-term efficacy and safety, and head-to-head data are lacking.

### 2.3 SYK inhibitors

SYK is an intracellular protein tyrosine kinase that participates in signalling for many immunological receptors, such as the Fcγ receptor, B cell receptor, and T cell receptor (Cooper et al., 2023). As a SYK inhibitor, fostamatinib reduces platelet destruction and has therapeutic benefits in ITP by blocking Fcγ-mediated signalling and inhibiting SYK activity via its active metabolite, R406 (Paik, 2021).

Gonzalez-Lopez et al. (2024) studied the efficacy and safety of fostamatinib in 138 patients with ITP, including both main and secondary types, who had already tried an average of 4 other treatments before starting Fostamatinib. According to the findings, 53.6% of patients achieved a CR (defined as a platelet count >100 × 10^9^/L), and 79.0% of patients reached the ORR (defined as a platelet count of ≥30 × 10^9^/L and a doubling from baseline). The proportion of patients with a platelet count of ≥50 × 10^9^/L was 71.7%. The most frequent AEs in 48.5% of patients were diarrhoea and hypertension, primarily in grades 1–2. The results of this real-world investigation indicate that fostamatinib has notable efficacy and favourable safety in individuals with primary or secondary ITP.

33 patients were included in the Phase III trial R788-1301 (NCT04132050) (Kuwana et al., 2024), which assessed the safety and effectiveness of fostamatinib in Japanese patients with primary ITP (Fostamatinib group, n = 22; Placebo group, n = 11). In addition, the investigators further evaluated the long-term effects of Fostamatinib over 52 weeks in patients with persistent or chronic ITP. In a prior double-blind trial, these patients received either Fostamatinib or a placebo. Patients who entered the long-term extension study continued to receive Fostamatinib. Compared to 14% of patients in the placebo arm, 43% of patients in the fostamatinib arm experienced at least one platelet count of ≥50,000/µL within the first 12 weeks of treatment. In long-term therapy, Fostamatinib demonstrated greater efficacy than placebo (18% vs. 2%), significantly enhancing and maintaining patients’ platelet counts for a longer duration. The two groups had similar rates of severe adverse events (13% in the Fostamatinib group and 21% in the placebo group), with the former experiencing more frequent occurrences of diarrhoea, hypertension, and dizziness. The long-term extension study demonstrated a positive safety profile for Fostamatinib, with no additional safety signals detected.

Sovleplenib (HMPL-523), an innovative SYK inhibitor, demonstrates therapeutic efficacy by suppressing SYK activity and obstructing FcγR-mediated signalling, hence diminishing autoantibody synthesis and reducing platelet disruption and phagocytosis by B cells and macrophages (Mingot-Castellano, 2024; Cai et al., 2024). 188 individuals were randomly assigned to the sovleplenib group (n = 126) and the placebo group (n = 62) in the phase III clinical investigation ESLIM-01 (NCT05029635) (Hu et al., 2024), conducted in China. With the main end objective being durable response rate, the purpose of the trial was to determine whether sovleplenib raises platelet counts and improves quality of life in adult patients with chronic primary ITP. The lasting response rate was 48% in the sovleplenib arm compared with 0% in the placebo arm. Compared with the placebo group, the sovleplenib group had a considerably greater cumulative number of weeks with platelet counts ≥50 × 10^9^/L (66.7 weeks vs. 50.4 weeks). The occurrence of TEAEs recorded in the two groups was 99% and 85%, respectively, and predominantly low grade. Frequent TEAEs of grade 3 or higher for sovleplenib versus placebo were platelet count decreased, neutrophil count decreased, and hypertension. The incidence of serious TEAEs was 21% in the sovleplenib group versus 18% in the placebo group. Previously, in a phase 1b/2 study (Liu et al., 2023), the sovleplenib 300 mg group had a much greater response rate than the placebo group (63% vs. 9%). The sustained response rate in the sovleplenib 300 mg group was 31%, compared with 0% in the placebo group. Based on the safety review, the researchers suggested that sovleplenib be given at 300 mg in phase 2. There were 34 cases of TEAEs in the sovleplenib group, most of which were low to moderate.

### 2.4 FcRn antagonists

To treat ITP, efgartigimod, a new humanised IgG1 Fc fragment, targets the neonatal Fc receptor, lowers circulating IgG antibody levels, and reduces platelet destruction, thereby raising platelet counts (Broome et al., 2023a).

A large Phase III study called ADVANCE IV (NCT04188379) (Broome et al., 2023a; Ma et al., 2024) examined how well and safely efgartigimod works in adults with primary ITP to determine if it can provide a lasting and effective increase in platelet counts. 131 patients were randomly assigned to the efgartigimod group (n = 86) and the placebo group (n = 45). The primary objective was a platelet count of ≥50 × 10^9^/L on at least 4 of 6 follow-up visits during weeks 19–24 of treatment, with no intervening events. During the first week of treatment, 38.4% (n = 33/86) of patients in the efgartigimod cohort attained a platelet count of 30 × 10^9^/L, whereas 11.1% (n = 5/45) in the placebo cohort did so. Additionally, compared to 6.7% (n = 2/45) in the placebo group, 22.1% (n = 19/86) of the patients in the efgartigimod group experienced a persistent response for at least 6 weeks. Malaise, headaches, and nausea were the most common TEAEs in the efgartigimod group, and they were mostly low to moderate. The rate of bleeding events was higher in the placebo group than in the efgartigimod group (86.7% vs. 70.9%). This finding implies that for patients with resistant ITP, efgartigimod presents a feasible therapy option.

However, because FcRn antagonists reduce pathogenic IgG antibodies, they exert their effects by binding to FcRn. Simultaneously, they transiently decrease all IgG subclasses, including protective neutralising antibodies, thereby posing a potential risk of infection. The guidelines explicitly state that the use of FcRn inhibitors requires attention to the resulting reduction in IgG levels and the potential risk of infection, emphasising the importance of preventive strategies (Neunert et al., 2019). Therefore, the implementation of protective measures is essential to ensure patient safety. Vaccination is crucial for bridging the protective antibody gap and establishing active immune barriers. Consequently, it is strongly recommended to complete the recommended vaccination schedule at least 4 weeks before initiating FcRn inhibitor therapy to ensure sufficient time for the body to generate adequate protective antibodies. Additionally, total IgG levels should be regularly monitored, patients should be counselled regarding periods of increased infection risk, the importance of early symptom recognition should be emphasised, and patients should be instructed to contact their healthcare team immediately upon the onset of any infectious signs.

### 2.5 Anti-CD38 monoclonal antibodies

The transmembrane glycoprotein CD38 is widely expressed on the surfaces of many immune cells, including macrophages, T cells, and plasma cells (Bocuzzi et al., 2024). By binding CD38 with high affinity and reducing autoantibody formation, CM313—a new humanised anti-CD38 monoclonal antibody—specifically identifies and targets these cells, therefore attenuating platelet loss in ITP patients (Liu W. et al., 2024).

The phase 1/2 experiment NCT05694767 (Chen et al., 2024a) assessed the safety and efficacy of CM313 in treating adult ITP, enrolling 22 patients who received weekly CM313 therapy (16 mg/kg) for 8 weeks. A platelet count of ≥50 × 10^9^/L was attained by 95.5% (n = 21/22) of patients during treatment, and at week 8, overall efficiency was 81.8% (n = 18/22). The proportion of patients with bleeding decreased significantly from 68.2% (n = 15/22) at baseline to 4.8% (n = 1/21) at week 8. Upper respiratory tract infections (32%) and infusion reactions (32%), with the majority being grade 1 or 2, were the most prevalent TRAEs. Furthermore, by eliminating antibody-secreting cells and preventing antibody-dependent cytotoxicity, the study demonstrated CM313’s dual mechanism of action, providing a new theoretical foundation for the treatment of ITP. In conclusion, CM313 demonstrated encouraging efficacy in this study, characterised by rapid platelet recovery. However, given the small sample size, these findings should be interpreted as preliminary and require validation in larger randomised controlled trials.

### 2.6 Anti-CD20 monoclonal antibodies

CD20 is a B-cell-specific antigen that plays an important role in B-cell formation and functional control (Friedberg, 2023). Zuberitamab effectively identifies and targets CD20-positive B cells, binds selectively to the CD20 antigen on their surfaces, and reduces autoantibody synthesis. A multicenter, phase II trial (ChiCTR2100050513) (Xu et al., 2024a) assessed the safety, effectiveness, and anticipated therapeutic dosage of zuberitamab in Chinese adult patients with primary ITP. Thirty-two patients were randomly divided into the placebo group (n = 4), the zuberitamab 100 mg group (n = 10), the zuberitamab 300 mg group (n = 8), and the zuberitamab 600 mg group (n = 10). According to the findings, by week eight of treatment, 40% of patients in the 100 mg dosage group had a platelet count of ≥50 × 10^9^/L. At week 12 of treatment, the percentages of patients with platelet counts ≥50 × 10^9^/L were 70% (n = 7/10) in 100 mg group, and those with counts ≥100 × 10^9^/L were 50% (n = 5/10) in 600 mg group. The trial concluded that the expected therapeutic dose of zuberitamab is 100 mg. Patients in all groups reported one or more AEs, the majority of which were minor or moderate and self-resolving. Infections, fever, and nutritional problems were the most prevalent AEs.

### 2.7 Complement inhibitors

Sutimlimab is a monoclonal antibody of the IgG4 subclass that binds to and stops complement protein C1S. This prevents the classical complement pathway from activating and reduces complement-mediated haemolysis, making it a therapeutic agent (Kapps et al., 2024; Gavriilaki et al., 2022). The Phase 1 study (NCT03275454) (Broome et al., 2023b) evaluated the efficacy, pharmacodynamics, and safety of biweekly sutimlimab in patients with chronic/refractory ITP. Twelve patients were enrolled, each receiving a median of 20.5 weeks of sutimlimab, followed by a median washout period of 2 weeks (Part A). In Part B, seven qualified patients received re-treatment for a median of 113 weeks. The mean platelet count increased rapidly after treatment, from 25 × 10^9^/L at baseline to 54 × 10^9^/L, and remained at ≥50 × 10^9^/L throughout Part A. Of the patients, 42% (n = 5/12) had a persistent platelet response (platelet count ≥50 × 10^9^/L), and 4 patients achieved a complete response (platelet count ≥100 × 10^9^/L). Patients’ average platelet counts returned to baseline during the washout period, and normalized quickly following re-treatment in Part B. There were 74 TEAEs in part A (n = 10) and 70 in part B (n = 6). The most frequently reported TEAE was fatigue. These findings provide preliminary evidence supporting the pathogenic role of the classical complement pathway in certain ITP patients. C1s inhibition therefore emerges as a promising therapeutic approach, although these results require confirmation in larger, multicentre, controlled studies.

### 2.8 JAK1/2 inhibitors

Janus kinase (JAK) is essential for signalling several growth factors and cytokines (Xue et al., 2023). Baricitinib is a selective inhibitor of JAK1 and JAK2, a class of JAK1/2 inhibitors, that effectively blocks the activity of these kinases and consequently inhibits downstream signalling pathways (McHugh, 2023; Lopez De Hontanar Torres et al., 2024). By blocking the JAK/STAT signalling pathway, baricitinib may reduce the development of autoantibodies in ITP, thereby alleviating patient symptoms. Baricitinib was evaluated in a phase 2 clinical trial (NCT05446831) to assess its safety and efficacy in people with glucocorticoid-resistant or recurrent ITP (Zhao et al., 2024). Of the 14 patients, 93% (n = 13/14) achieved platelet counts ≥30 × 10^9^/L and a more than two-fold increase from baseline after treatment, with 71% (n = 10/14) reaching ≥50 × 10^9^/L. During a median 12-month follow-up, 10 of 13 patients maintained a platelet count ≥ 50 × 10^9^/L. During the safety evaluation, 13 patients experienced at least 1 AE, predominantly mild in severity. These findings indicate that baricitinib may serve as a viable second-line treatment option for ITP.

## 3 Cell therapies

Refractory ITP is characterised by patients who have not responded to or relapsed on numerous available treatments (Cines, 2023). The effectiveness and safety of human umbilical cord-derived mesenchymal stem cells (UC-MSCs) in treating refractory ITP were assessed in a phase 1 trial (NCT04014166) (Chen et al., 2024b), which included 18 patients who received weekly UC-MSCs at different dose levels (0.5 × 106 cells/kg, 1.0 × 106 cells/kg, and 2.0 × 106 cells/kg). The findings indicated an overall response rate of 44.4% (n = 8/18), with all patients in the 2.0 × 10^6^ cells/kg dosage group attaining a platelet count of around 50 × 10^9^/L, which was sustained for 28 weeks 12 patients and 6 patients were included in the dose-escalation and dose-expansion phases, with response rates of 41.7% (n = 5/12) and 50.0% (n = 3/6), respectively. 60.0%–75.0% of patients had complete remission of bleeding symptoms after treatment. No severe TEAEs were reported, and 72.2% of patients (n = 13/18) had one or more TEAEs, the majority of which were low grade. Furthermore, the researchers found that UC-MSCs may also modulate immune cell ratios, potentially aiding in remodelling the immunological milieu in ITP patients. This research offers the first evidence supporting the use of UC-MSCs for treating refractory ITP, necessitating larger multicenter trials in the future to confirm their long-term efficacy and safety.

The single-pass transmembrane protein GPIb α is crucial for platelet adhesion, haemostasis, immunological response, and thrombopoiesis (Yan R. et al., 2024). It binds to filamin A via its cytoplasmic tail. Abnormal GPIbα function is linked to several bleeding diseases, such as anti-GPIbα antibodies in ITP, which can destroy platelets and disrupt haemostasis (Ellis et al., 2024). GPIbα is an important autoantigen in ITP and is strongly correlated with treatment resistance. Han et al. (2025) assessed the efficacy of CD19 CAR-T cell treatment in a modified murine model of GPIbα. The findings showed that mice treated with CD19 CAR-T cells had higher platelet counts more quickly than the control group. Furthermore, CD19 CAR-T cells efficiently depleted CD19^+^ B cells and CD138^+^ plasma cells and markedly diminished anti-GPIbα autoantibodies. No significant cytokine release syndrome was observed in the treated mice, suggesting a favourable safety profile for the therapy. *In vitro*, CD19 CAR-T cells demonstrated potent cytotoxic activity against CD19^+^ B cells and CD138^+^ plasma cells, the key autoantibody-producing cells in ITP pathogenesis. The aforementioned findings provide a useful point of reference for the potential use of CD19 CAR-T cell therapy in ITP.

## 4 Other emerging therapies

In the pathophysiology of ITP, macrophages play a significant role. By directly engaging with the endoplasmic reticulum stress response, YC-4-3, a new inhibitor of glycogen synthase kinase 3β (GSK 3β), can improve thrombocytopenia in ITP and regulate the inflammatory state of macrophages. Xu et al. (2025) used both basic and clinical investigations to confirm the effects of YC-4-3 on macrophages in ITP. GSK 3β-positive cells were elevated in monocytes from individuals with newly diagnosed ITP and diminished in those who responded to treatment. In addition, YC-4-3 demonstrated the ability to decrease pro-inflammatory differentiation, phagocytosis, and cytokine generation in macrophages while simultaneously mitigating thrombocytopenia in ITP. This may be related to YC-4-3’s ability to inhibit the endoplasmic reticulum stress signalling pathway. In animal experiments, YC-4-3 significantly increased platelet counts and alleviated thrombocytopenia symptoms in ITP model mice. This study indicates that YC-4-3 may be a potential treatment for ITP; however, its efficacy and safety in clinical patients require further validation.

Sirolimus, a mammalian target of rapamycin inhibitor, has potential for treating refractory ITP through its unique immunomodulatory mechanism, which regulates T cell function and promotes regulatory T cell differentiation. Currently, international consensus guidelines do not include it in standard treatment pathways. It is typically considered only for refractory or relapsed ITP patients when other second-line therapies prove ineffective or are not tolerated. A study involving 30 hormone-dependent ITP patients explored the role of Sirolimus combined with hormone reduction regimens in patients (Lv et al., 2022). Results demonstrated an overall response rate of 83.3% (n = 25/30) and a complete response rate of 60% (n = 18/30), with all patients successfully discontinuing corticosteroids. This study preliminarily confirms the potential of Sirolimus in treating ITP. However, current research on Sirolimus is predominantly single-centre and small-sample in scope, lacking large-scale Phase III randomised controlled trials. This prevents further confirmation of its precise efficacy in ITP. Therefore, future studies could involve large-scale phase 3 randomised controlled trials to clarify its effectiveness and safety in refractory ITP. Additionally, investigations exploring the combination of Sirolimus with other drugs for treating ITP are also warranted.

**FIGURE 2:**
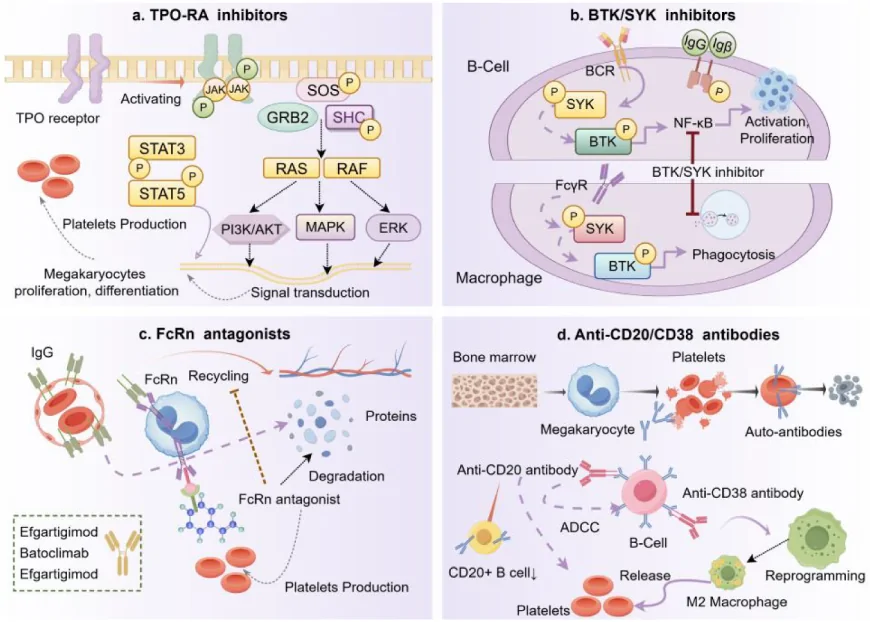
The main targeted therapeutic mechanisms for primary immune thrombocytopenia. Schematic illustration of major targeted therapeutic mechanisms for primary immune thrombocytopenia. **(a)** TPO-RA inhibitors: Thrombopoietin receptor agonists bind to the TPO receptor on megakaryocytes, activating downstream signaling cascades including JAK-STAT, PI3K-AKT, and MAPK-ERK pathways. This promotes megakaryocyte proliferation, differentiation, and ultimately increases platelet production. **(b)** BTK/SYK inhibitors: Bruton’s tyrosine kinase inhibitors block B-cell receptor signaling, while spleen tyrosine kinase inhibitors interfere with Fcγ receptor-mediated activation. Both reduce autoantibody generation and macrophage-mediated platelet destruction. Key downstream mediators (e.g., RAS, RAF, GRB2, SHC, SOS) are also indicated. **(c)** FcRn antagonists: Neonatal Fc receptor antagonists prevent the recycling of IgG antibodies, directing them toward lysosomal degradation. This lowers circulating pathogenic autoantibody levels, thereby reducing platelet clearance. **(d)** Anti-CD20/CD38 antibodies: Monoclonal antibodies targeting CD20 (on B cells) and CD38 (on plasma cells) deplete these autoantibody-producing cells via antibody-dependent cellular cytotoxicity and other effector mechanisms, decreasing autoantibody secretion and improving platelet counts. Abbreviations: TPO-RA, Thrombopoietin receptor agonists; JAK, Janus kinase; STAT, Signal Transducer and Activator of Transcription; SOS, Son of Sevenless; GRB2, Growth Factor Receptor-Bound Protein 2; SHC, Src Homology 2 Domain-Containing Transforming Protein; RAS, Rat Sarcoma Virus; RAF, Rapidly Accelerated Fibrosarcoma; PI3K/AKT, Phosphoinositide 3-Kinase/Protein Kinase B; MAPK, Mitogen-Activated Protein Kinase; ERK, Extracellular Signal-Regulated Kinase; BTK, Bruton’s Tyrosine Kinase; SYK, Spleen Tyrosine Kinase; NF-κB, uclear Factor-kappa B; FcRn, Neonatal Fc Receptor; ADCC, Antibody-Dependent Cell-Mediated Cytotoxicity.

KB-208 is a new small-molecule inhibitor that primarily increases ITP by preventing macrophage phagocytosis (Loriamini et al., 2023). In a mouse model of ITP, Loriamini et al. (2024) investigated the possible therapeutic benefits of KB-208 and contrasted it with intravenous immunoglobulin (IVIG). Three different mouse strains (BALB/c, C57BL/6, and CD1) were used for comparison. Across all three mouse strains, KB-208 demonstrated an efficacy comparable to that of IVIG. No substantial *in vivo* toxicity was observed in CD1 mice following chronic treatment with KB-208 at a dose of 6 mg/week for 60 days Table 1 shows several ongoing studies of novel drugs for ITP patients. Figure 2 shows the main targeted therapeutic mechanisms for primary immune thrombocytopenia.

**TABLE 1:** Several ongoing studies of novel drugs for ITP patients^a^.

Number	Drug name	Enrollment	Primary end points	Secondary end points	Phase
NCT06497036	GP40141	160	platelet response, AEs, Proportion of subjects experiencing AEsetc.	Not reported	3
NCT06004856	Orelabrutinib	195	DRR	Not reported	3
NCT06291415	HMPL-523	48	AEs, DLT	Cmax, Tmax, Cmin, etc	1
NCT04812925	Efgartigimod PH20	173	AEs, SAEs, ECGetc.	Extent of disease control, overall platelet count responseetc.	3
NCT06544499	Efgartigimod IV	63	Extent of disease control	Time to respond, Proportion of participants with initial responseetc.	3
NCT06594146	CM313	62	TEAEs, SAEs	Not reported	2
NCT04669600	BIVV020	12	Durable Platelet Response	TEAEs, SAEsetc.	2
NCT05852847	Baricitinib	216	Durable response	CR, Initial response, AEsetc.	2
NCT05932524	Baricitinib	132	Durable response	CR, DOR, AEsetc.	2
NCT05338190	Belimumab	132	ORR	Platelet levels, Number of severe infectionsetc.	3
NCT06787989	BCMA-CD19 cCAR-T	20	AEs	ORR	1

^a^ AEs, Adverse events; SAEs, Serious adverse events; TEAEs, Treatment-emergent adverse events; DRR, durable response rate; ORR, overall response rate; DLT, dose limiting toxicities; Cmin, minimum plasma drug concentration; Cmax, maximum plasma drug concentration; Tmax, time to reach maximum plasma drug concentration. ECG, electrocardiogram; CR, complete response; DOR, duration of response; ORR, overall response rate; TTF, time from randomisation to treatment failure; BCMA, B-cell maturation antigen; cCAR, compound chimeric antigen receptor.

## 5 Combination therapy strategies

Eltrombopag, a thrombopoietin receptor agonist, increases platelet production by preferentially binding to the TPO-R transmembrane domain and activating the TPO signalling pathway, which in turn encourages megakaryocyte proliferation and differentiation (Subbarayan et al., 2024). In addition to directly promoting platelet formation, eltrombopag has immunomodulatory effects, raising the amount of regulatory CD4^+^ T cells while decreasing the function of effector T cells (Drexler and Passweg, 2021).

In patients with primary ITP who did not respond to full-dose eltrombopag for 14 days, a Phase 2 study (NCT04917679) investigated the safety and efficacy of eltrombopag plus diacerein compared with eltrombopag alone (Sun et al., 2024). The researchers postulated that diacerein might act as a sensitiser, enhancing eltrombopag’s effectiveness. The trial included 102 eligible patients, who were randomly assigned to two groups: the combination group (n = 50) and the monotherapy group (n = 52). In the combination group, the total remission rate was 44% after 15 days of treatment, while in the monotherapy group, it was only 13%. Remission is defined as a platelet count of 30 × 10^9^/L or above, at least twice the baseline level, and no bleeding. Furthermore, the reaction duration in the combination arm was much longer than in the eltrombopag arm. The incidence of AEs was similar in both groups. In the combination group, gastrointestinal reactions and respiratory infections were the most common TEAEs, while in the eltrombopag group, exhaustion and respiratory infections were the most common. Combination therapy significantly improves overall remission rates and is well tolerated by patients. This makes it an effective salvage treatment option, ensuring patients do not have to switch to other, less effective therapies quickly.

The cytokine B-cell activating factor (BAFF), a member of the tumour necrosis factor family, is essential for B-cell survival, maturation, and function. In autoimmune diseases, BAFF levels are often elevated, leading to B-cell hyperactivation and the production of autoantibodies (Cheekati and Murakhovskaya, 2024). The completely human monoclonal antibody belimumab blocks the binding of soluble BAFF to the B-cell receptor. This prevents B cells from transforming into plasma cells, hence halting the generation of autoantibodies and improving the symptoms of ITP sufferers (Singh et al., 2021; Jiang et al., 2022). By effectively decreasing the frequency of CD20-positive B cells in lymphoid tissues and peripheral blood, the monoclonal antibody rituximab inhibits the generation of autoantibodies and the overactivation of the immune response (Xiao and Murakhovskaya, 2023). Rituximab is now widely used to treat several B-cell-related disorders, including ITP and systemic lupus erythematosus. Certain studies indicate that combining an anti-BAFF antibody with an anti-CD20 antibody can markedly enhance therapeutic efficacy in individuals with ITP. The RITUX-PLUS trial (NCT03154385) (Mahevas et al., 2021), for instance, showed that the rituximab plus belimumab group was well tolerated and had higher overall and response rates than the Rituximab group. This study provides valuable preliminary evidence supporting the use of rituximab in combination with belimumab for the treatment of ITP; however, as a small, single-arm exploratory trial, its findings still need to be validated by larger, more rigorously designed randomized controlled trials.

By specifically binding to BAFF-R, inhibiting BAFF’s interaction with its receptor, and reducing B-cell overactivation and survival, ianalumab (VAY736), a humanised monoclonal antibody against the BAFF receptor (BAFF-R), exhibits therapeutic potential in a range of autoimmune diseases (Nilforoushzadeh et al., 2024).

The VAYHIT1 (NCT05653349) trial aims to assess the efficacy and safety of ianalumab compared to placebo in adult patients with primary ITP receiving first-line glucocorticoids. The trial is now underway, and the findings are expected to bring novel ideas and approaches to the treatment of primary ITP. Another Phase 3 clinical study, VAYHIT2 (NCT05653219), aims to determine whether ianalumab (VAY736), when used in combination with the thrombopoietin receptor agonist eltrombopag, prolongs time to treatment failure in patients with primary ITP who have not responded to or relapsed on first-line glucocorticoid therapy, compared with placebo. The period from randomisation to treatment failure—defined as a platelet count below 30G/L, the need for rescue medication, the initiation of new ITP therapy, the inability to discontinue or reduce eltrombopag, or death—was the main outcome of the study. The experiment is currently recruiting participants, and its outcomes will yield significant data regarding the application of ianalumab in ITP.

Neuraminidase is an enzyme that hydrolyzes the terminal Sialic acid of glycoproteins (Xu J. et al., 2024). In ITP, after sialic acid on the surface of platelets is removed by neuraminidase, the platelets are identified and cleared by Ashwell-Morell receptors in the liver (Zheng et al., 2022). Neuraminidase inhibitors (such as oseltamivir) reduce the desialylation of platelets by inhibiting the activity of neuraminidase, thereby prolonging the lifespan of platelets and reducing platelet clearance (Wang et al., 2023). A phase II study (NCT01965626) (Sun et al., 2021) enrolled 96 adult ITP patients, who were randomly assigned to receive either dexamethasone alone (monotherapy group) or dexamethasone combined with oseltamivir (combination group) to assess whether dexamethasone combined with oseltamivir is more effective than dexamethasone alone. The rate of sustained response at 6 months after treatment was 53% (n = 23/47) in the combination group and 30% (n = 14/49) in the monotherapy group, which was significantly higher in the combination group than in the monotherapy group. In addition, the 14-day response rate was significantly higher in the combination group versus the monotherapy group (86% vs. 66%). The safety profile was similar in the two groups, and no serious AEs related to oseltamivir were observed. AEs in the monotherapy group were mainly glucocorticoid side effects, while those in the combination group were mainly mild gastrointestinal reactions. In conclusion, the combination of dexamethasone and oseltamivir in the treatment of newly diagnosed ITP patients can more quickly increase platelet counts and maintain higher platelet levels for a longer period of time, with favorable safety. These results support the conduct of larger clinical trials in the future to further validate the safety and efficacy of combination therapy. Table 2 presents the treatment options for adult patients with ITP.

**TABLE 2:** The treatment options for adult patients with ITP^a^.

Therapeutic phase	Treatment strategy	Indicated populations	Recommendation level	References
First-line	Short-term corticosteroids (prednisone ≤8 weeks, or dexamethasone pulse therapy)	Newly diagnosed ITP with platelet count <20–30 × 10^9^/L or high bleeding risk	The guidelines explicitly recommend (Grade A)	Neunert et al. (2019), Provan et al. (2019)
First-line	IVIG	Newly diagnosed ITP	The guidelines explicitly recommend (Grade A)	Neunert et al. (2019), Provan et al. (2019)
First-line	Corticosteroids plus IVIG	Patients with severe bleeding or high-risk bleeding require rapid platelet elevation	The guidelines explicitly recommend (Grade A)	Provan et al. (2019)
First-line	Dexamethasone plus Rituximab	Newly diagnosed ITP	Considered for selected patients (Grade B)	Gudbrandsdottir et al. (2013), Zaja et al. (2010)
First-line	Dexamethasone plus MMF	Newly diagnosed ITP	Considered for selected patients (Grade B)	Bradbury et al. (2021)
First-line	Dexamethasone plus rhTPO/TPO-RA	Newly diagnosed ITP	Research stage (Grade C)	Zhang et al. (2020)
First-line	Dexamethasone plus Oseltamivir/ATRA	Newly diagnosed ITP	Research stage (Grade C)	Neunert et al. (2019)
Second-line	TPO-RAs	Patients who are unresponsive to, dependent on, or experience recurrence with corticosteroids	The guidelines explicitly recommend (Grade A)	Neunert et al. (2019)
Second-line	Rituximab	Patients seeking limited-course treatment, especially younger patients	The guidelines explicitly recommend (Grade A)	Provan et al. (2019)
Second-line	Fostamatinib	TPO-RA or rituximab contraindicated/ineffective	The guidelines explicitly recommend (Grade A)	Gonzalez-Lopez et al. (2024)
Second-line	TPO-RA plus Immunosuppressants	For those who respond poorly to a single drug	Considered for selected patients (Grade B)	Neunert et al. (2019)
Second-line	Rituximab plus rhTPO/ATRA	Hormone-resistant/relapsed individuals	Considered for selected patients (Grade B)	Wu et al. (2022)
Third-tier and above	TPO-RA plus Immunosuppressants	Multiple refractory ITP (unresponsive to ≥2 second-line therapies)	Considered for selected patients (Grade B)	Mahevas et al. (2016)
Third-tier and above	Other immunosuppressant(MMF, Ciclosporin, Danazol, etc.)	Patients with multidrug resistance	Considered for selected patients (Grade B)	Moulis et al. (2024)
Third-tier and above	Splenectomy	Patients for whom drug therapy has been ineffective and who are willing to undergo surgery	The guidelines explicitly recommend (Grade B)	Godeau (2023)
Third-tier and above	Daratumumab	Multiple refractory ITP	Research stage (Grade C)	Tsykunova et al. (2026)
Third-tier and above	New drugs such as decitabine	Refractory ITP	Research stage (Grade C)	(Han et al., 2021)
Specific populations	Preferred IVIG and/or corticosteroids	Pregnant individuals	The guidelines explicitly recommend (Grade A)	Bussel and Knightly (2024)
Specific populations	Prioritise rituximab or Fostamatinib; avoid TPO-RA	Patients at high risk of thrombosis (including those with antiphospholipid antibodies or a history of thrombosis)	The guidelines explicitly recommend (Grade A)	Bussel et al. (2018)
Specific populations	Consider combination therapy or multi-targeted drugs, prioritising oral formulations	Refractory ITP with multiple complications	Personalized treatment	Al-Samkari and Neufeld (2023)
Specific populations	Elderly individuals/those at risk of infection	Avoid long-term corticosteroids; TPO-RA or Fostamatinib may be considered	Personalized treatment	Crickx et al. (2023)

^a^ ITP, immune thrombocytopenia; IVIG, intravenous immunoglobulin; MMF, mycophenolate mofetil; TPO-RA, thrombopoietin receptor agonists; ATRA, All-trans-retinoic acid; Grade A, Requires ≥1 randomised controlled trial (RCT) as part of a body of literature of overall good quality and consistency addressing the specific recommendation (evidence levels Ia, Ib); Grade B, Requires the availability of well-conducted clinical studies but no randomised clinical trials on the topic of recommendation (evidence levels IIa, IIb, III); Grade C: Requires evidence obtained from expert committee reports or opinions and/or clinical experiences of respected authorities, indicating an absence of directly applicable clinical studies of good quality (evidence level IV).

## 6 Biomarkers and response prediction

Traditional autoantibody testing holds a fundamental position in the diagnosis of ITP, but its limitations have driven research toward more refined cellular and molecular levels.

### 6.1 Traditional predictive indicators

Autoantibodies against platelet surface glycoproteins (such as GPIIb/IIIa, GPIb/IX) are a classic marker of ITP (Chatterjee, 2022). However, due to both their sensitivity and specificity not being ideal, they are currently mainly used to support diagnostic indicators rather than confirmatory indicators. About 25%–39% of ITP patients tested positive for anti-nuclear antibodies (ANA), which may have some predictive value. A study indicates that ANA-positive patients may exhibit better early responses to rituximab but potentially poorer long-term outcomes, and this may be related to suboptimal responses to eltrombopag therapy (Liang et al., 2025; Pan et al., 2025). This suggests that ANA status may be valuable for preliminary prediction of drug selection preferences.

The Immature Platelet Fraction (IPF) reflects the release of newly generated platelets from the bone marrow (Zhang et al., 2025). In peripheral destruction disorders such as ITP, the IPF is typically elevated as a compensatory response, whereas it tends to be decreased in bone marrow production disorders. Therefore, IPF is an important diagnostic tool for clinically effective differentiation of thrombocytopenia mechanisms (Asghar et al., 2023). Research has revealed that refractory ITP patients may exhibit expanded T-cell clones with abnormal characteristics, which could explain treatment failure (Ji et al., 2022). TCR sequencing and gene expression profiling have revealed preliminary associations with clinical features and treatment responses in retrospective studies, suggesting their potential as exploratory tools for ITP subtyping. However, prospective clinical validation in independent cohorts is required before these approaches can be considered for routine clinical application.

### 6.2 Novel predictive indicators

Multiomics technologies are profoundly changing our understanding of the complex immune dysregulation mechanism of ITP, and discovering many new potential biomarkers. Research in 2024 confirmed that the C-X-C motif ligand 13/C-X-C Motif Chemokine Receptor 5 signaling axis plays a crucial role in driving the expansion of follicular helper T cells (Chen Z. et al., 2024). Increased activity of this axis was significantly associated with lower platelet count and poorer treatment response, making it a potential marker of disease severity and treatment response. Moreover, studies have characterized the immune cell heterogeneity in the bone marrow of ITP patients at the single-cell level. The results revealed that the expression of co-inhibitory molecules such as cytotoxic T lymphocyte-associated antigen-4 was significantly reduced on Th1 and Th17 cell subsets. This high-dimensional proteomic map not only reveals disease mechanisms with greater precision but also offers new insights for developing personalized immunotherapies targeting these immune checkpoints (Liu F. et al., 2024).

Genetic polymorphism analysis offers perspectives for understanding the risk factors associated with ITP. For instance, individuals carrying the TNF-α-857 TT genotype and the MCP1^+^2518 G allele exhibit a significantly increased risk of developing chronic ITP. Such genetic markers contribute to identifying high-risk populations (Xu et al., 2024b; Wang et al., 2014; Zhang et al., 2019).

## 7 Potential issues

Currently, significant gaps still exist in the treatment plans and diagnostic techniques for ITP. We believe that the following are the primary factors

1. Presently, the diagnosis of ITP is primarily based on the method of exclusion, which necessitates the exclusion of other conditions that may induce thrombocytopenia, including bone marrow disorders, pharmacologic thrombocytopenia, and chemotherapy-induced thrombocytopenia. This process is time-consuming and increases the likelihood of misdiagnosis. Potential markers such as microRNAs (Garabet et al., 2025), genes linked to metabolism, and inflammatory factors have been suggested, but their specificities and sensitivities are still below the level required for clinical application,
2. The bleeding risk in ITP patients does not exhibit a completely linear correlation with platelet count; some individuals may present no apparent bleeding symptoms despite having significantly low platelet levels. Since there are currently no trustworthy biomarkers or scoring methods to precisely estimate bleeding risk, it is challenging to decide when to implement clinical interventions (Oshinowo et al., 2024).
3. Special populations of ITP patients, such as youngsters and pregnant women, are currently receiving less attention.
4. There is a greater need for clinical attention to individualised treatment because of the notable variances in patient response to the same medicine, which may be related to genetic background, autoantibody subtype (anti-GPIIb/IIIa vs. GPIb/IX), or the proportion of immune cell subpopulations.
5. Despite advancements with novel targeted agents, including TPO-RA, BTK, and SYK inhibitors, certain patients continue to experience low response rates or treatment resistance. Additionally, patients’ use of the new medications is somewhat restricted by their high cost and lengthy clinical trial duration. Current international ITP treatment guidelines are mainly based on evidence from resource-rich regions, which leads to their limited applicability globally. In resource-poor regions, even when new medicines are available, the high costs and complex medical infrastructure requirements mean that traditional treatments like splenectomy remain a reluctant yet practical choice. Moreover, cutting-edge drugs such as BTK inhibitors, novel SYK inhibitors, and FcRn inhibitors are highly concentrated in developed countries or major cities, leaving patients in impoverished regions and smaller cities unable to benefit. The high cost of new therapeutic agents needs to be balanced against the long-term potential for lasting relief, significant improvements in quality of life, reduced side effects, and potentially reduced consumption of medical resources. Patient-centered care requires shared decision-making. However, when treatment options are limited in advance due to economic or geographic constraints, the decision-making process between doctors and patients cannot be truly equitable.

## 8 Potential solutions

Consequently, in light of the aforementioned issues, we assert that the subsequent solutions require meticulous consideration:

1. Screening for ITP-specific indicators by integrating multi-omics methods (such as transcriptomics, metabolomics, and single-cell sequencing). For instance, this approach can assist in diagnosing and staging patients based on their autoantibody profile, gut microbiome characteristics, and genetic attributes (Schoenaker et al., 2025).
2. To investigate the synergy of new and conventional targeted medications and to overcome the limitations of monotherapies.
3. Integrating platelet function assays, vascular endothelial biomarkers, and clinical attributes (including haemorrhagic history and comorbidities) to provide a dynamic risk assessment instrument for personalised intervention thresholds. For example, incorporating the ITP Bleeding Scale (IBLS). IBLS is suitable for quantitative assessment of hemorrhaging outcomes in clinical trials, objectively reflecting the efficacy of treatment in ameliorating hemorrhaging symptoms. Although IBLS was not classified as the preferred standardization tool in the ASH 2019 and international consensus guidelines, it was incorporated as a secondary endpoint in clinical trials such as Phase III LUNA, with its clinical utility validated by high-level research (Al-Samkari et al., 2021). Additionally, the ITP Bleeding Assessment Tool (ITP-BAT) is recommended by the ASH 2019 guidelines as a standardized bleeding assessment tool specifically for ITP. It can be used in clinical practice for individualized assessment of bleeding risk and treatment decision-making. However, the ITP-BAT relies on patient self-reporting or physician retrospective assessment, which introduces significant subjectivity (Beyene et al., 2023). Furthermore, its grading criteria for minor bleeding are ambiguous, leading to inconsistent evaluations across different medical centers. Moreover, certain biomarkers currently in the experimental stage or not explicitly recommended by guidelines also hold potential for bleeding risk stratification. For example, platelet function activation markers (P-selectin, PAC-1, reactive oxygen species ROS), inflammation-related ratios (platelet-to-lymphocyte ratio and hemoglobin-to-platelet ratio), and mean platelet volume can also serve as indicators for assessing bleeding risk in ITP patients (Nakhaei Shamahmood et al., 2025; Walle et al., 2023). Therefore, combining multiple bleeding assessment tools together may improve the accuracy of bleeding risk prediction.
4. To enhance the management of lifestyle issues and problems, and to provide integrated care for specific patient populations with ITP, like pregnant women or children, interdisciplinary teams are being developed. Pregnant women should prioritize treatment with IVIG and/or corticosteroids, while immunosuppressants such as mycophenolate mofetil, which have established teratogenicity, are contraindicated. However, corticosteroids often cause multiple adverse reactions, while IVIG imposes a significant treatment burden on patients due to its intravenous administration. Therefore, future efforts could focus on developing oral, low-cost immunomodulators to replace IVIG for patients who do not respond to steroids. At the same time, establishing biomarkers for ITP during pregnancy, such as PAIgG subtypes, would help enable individualized treatment decisions and reduce ineffective therapies. Older ITP patients are prone to both infection and high thrombotic risk, creating a dilemma in treatment decision-making. Thus, the development of ITP risk stratification tools for the elderly (integrating age, comorbidities, thrombosis/infection history), balancing efficacy and safety, and providing better guidance for drug selection (David et al., 2021). The pathological mechanism of COVID-associated ITP remains unclear, and there is currently no standardised treatment approach. Therefore, it is necessary to conduct research on its disease mechanisms, clarify the relationship between the virus and autoantibodies, and develop targeted therapies.
5. To lower treatment costs and encourage the development of new medications so that more ITP sufferers have access to options and medications. Given limited resources, reference guidelines should establish tiered treatment pathways suitable for local conditions. For instance, position cost-effective therapies with potential for sustained remission appropriately to provide viable alternatives for patients unable to afford novel drugs. Also, using digital platforms to remotely monitor and educate patients, improve treatment compliance, identify complications early, reduce unnecessary outpatient visits, thereby reducing the overall healthcare burden, and enable patients in remote areas to receive expert guidance. Simultaneously, physicians must thoroughly discuss all available treatment options with patients, including evidence of efficacy, potential side effects, financial burden, and impact on daily life, to ensure that treatment choices align with the patient’s personal values and life circumstances. The ultimate goal of these measures is to transform the “patient-centered” concept into high-quality, affordable healthcare services that are accessible to all ITP patients.

## 9 Summary and future perspectives

In conclusion, the treatment of primary immune thrombocytopenia has advanced considerably with the emergence of TPO-RAs, BTK inhibitors, SYK inhibitors, FcRn antagonists, and monoclonal antibodies targeting CD38, CD20, and complement components. Among these, TPO-RAs possess the strongest evidence base and are established as standard second-line therapy. BTK and SYK inhibitors have demonstrated reproducible efficacy in phase II/III trials, while antibody-based therapies show early promise but require larger confirmatory studies. Combination regimens and cell-based approaches remain investigational. Future research should prioritise comparative effectiveness trials, biomarker-driven patient stratification, and long-term safety surveillance to translate these pharmacological advances into personalised, evidence-based clinical practice.
